# Comparison of dementia recorded in routinely collected hospital admission data in England with dementia recorded in primary care

**DOI:** 10.1186/s12982-016-0053-z

**Published:** 2016-10-28

**Authors:** Anna Brown, Oksana Kirichek, Angela Balkwill, Gillian Reeves, Valerie Beral, Cathie Sudlow, John Gallacher, Jane Green

**Affiliations:** 1 Cancer Epidemiology Unit, Nuffield Department of Population Health, University of Oxford, Richard Doll Building, Roosevelt Drive, Oxford, OX3 7LF UK; 2 Centre for Clinical Brain Sciences, University of Edinburgh, Edinburgh, UK; 3 Department of Psychiatry, University of Oxford, Oxford, UK

**Keywords:** Dementia, Hospital Episode Statistics, Clinical Practice Research Datalink, Electronic health record, Cohort studies

## Abstract

**Background:**

Electronic linkage of UK cohorts to routinely collected National Health Service (NHS) records provides virtually complete follow-up for cause-specific hospital admissions and deaths. The reliability of dementia diagnoses recorded in NHS hospital data is not well documented.

**Methods:**

For a sample of Million Women Study participants in England we compared dementia recorded in routinely collected NHS hospital data (Hospital Episode Statistics: HES) with dementia recorded in two separate sources of primary care information: a primary care database [Clinical Practice Research Datalink (CPRD), n = 340] and a survey of study participants’ General Practitioners (GPs, n = 244).

**Results:**

Dementia recorded in HES fully agreed both with CPRD and with GP survey data for 85% of women; it did not agree for 1 and 4%, respectively. Agreement was uncertain for the remaining 14 and 11%, respectively; and among those classified as having uncertain agreement in CPRD, non-specific terms compatible with dementia, such as ‘memory loss’, were recorded in the CPRD database for 79% of the women. Agreement was significantly better (p < 0.05 for all comparisons) for women with HES diagnoses for Alzheimer’s disease (95 and 94% agreement with any dementia for CPRD and GP survey, respectively) and for vascular dementia (88 and 88%, respectively) than for women with a record only of dementia not otherwise specified (70 and 72%, respectively). Dementia in the same woman was first mentioned an average 1.6 (SD 2.6) years earlier in primary care (CPRD) than in hospital (HES) data. Age-specific rates for dementia based on the hospital admission data were lower than the rates based on the primary care data, but were similar if the delay in recording in HES was taken into account.

**Conclusions:**

Dementia recorded in routinely collected NHS hospital admission data for women in England agrees well with primary care records of dementia assessed separately from two different sources, and is sufficiently reliable for epidemiological research.

**Electronic supplementary material:**

The online version of this article (doi:10.1186/s12982-016-0053-z) contains supplementary material, which is available to authorized users.

## Background

Dementia is known to have a long pre-clinical phase [1, 2]. Large prospective cohort studies with long-term follow-up through linkage to routinely-collected hospital admissions records provide important opportunities for epidemiological investigations of dementia. The reliability of diagnoses of dementia in hospital data is, however, not well documented.

In the Million Women Study cohort, virtually complete follow-up for hospital admissions has been established by record linkage to routinely collected National Health Service (NHS) databases in England (Hospital Episode Statistics, HES) and Scotland (Scottish Morbidity Records). The linked hospital records contain coded diagnostic information for all inpatient and day-case admissions, and have been shown in this cohort to be reliable for ascertainment of vascular disease [3]. Primary care data, which is the most comprehensive single source of NHS information on consultations, prescriptions, diagnoses, treatments and referrals is held by each individual’s General Practitioner. Over 99% of the UK population is registered with a GP in the NHS [4]. The Clinical Practice Research Datalink (CPRD) has for many years collected coded information from GPs on diagnoses, prescriptions and other factors in primary care, with active coverage of around 7% of the UK population [5, 6].

For a sample of Million Women Study participants in England, we aimed to compare information on dementia recorded in hospital admission data (HES) with information on dementia obtained from two different sources of primary care data: (1) through linkage to coded CPRD records; and (2) postal survey information from a sample of study participants’ GPs.

## Methods

The Million Women Study (www.millionwomenstudy.org) has been described elsewhere [7, 8]. Between 1996 and 2001, over 1.3 million UK women aged 50–64 years were recruited through NHS breast screening programmes in England and Scotland. Women in the study gave written consent to follow-up through their NHS records. Linkages to routinely collected NHS records are done by matching women using their unique NHS number, together with other identifying details including date of birth and postcode. Follow-up for deaths is up to 31/12/2014 and, at that time, only 1% had been lost to follow up.

Electronically linked hospital admissions data from HES for the period 1 April 1997–31 March 2011 for the 1.25 million women recruited in England were provided by the Health and Social Care Information Centre (HSCIC) [9]. The HES records include admission and discharge dates and coded diagnostic data for any number of clinical conditions. Diagnostic data are routinely extracted from hospital medical records and coded by trained NHS clinical coders using the 10th Revision of the International Classification of Diseases (ICD-10 [10]). For this study, dementia in HES records and in death certificates was defined as any of the following ICD-10 codes: E512, F00, F01, F02, F03, F10.6, F10.7, G30, or G31.0. Some analyses were restricted to codes for Alzheimer’s dementia (ICD-10: F00, G30), vascular dementia (ICD10: F01) and dementia, not otherwise specified (NOS; ICD 10: F03).

The CPRD is a computerised UK research database containing linked anonymised patient records for patients registered with an NHS GP. Active coverage is around 7% of the UK population, with research-useable data available for some 11 m people [5, 6]. Records are coded by the individual’s GP using the Read code system. The database consists of longitudinal medical records with varying periods of observation, depending on when each individual joins or leaves a GP who contributes data to CPRD. Linked coded CPRD records for Million Women Study participants for the period 1 January 1990–31 December 2012 were provided by the CPRD division of the Medicines and Healthcare products Regulatory Agency (MHRA), with data linkage performed by HSCIC. Dementia in CPRD was defined here as any of 97 specific Read clinical codes and/or as a code for a drug specifically prescribed for dementia, i.e. donepezil, galantamine, memantine and rivastigmine (Additional file 1: Code list 1).

A further 92 Read codes (Additional file 1: Code list 2) that we considered compatible with, but not sufficient to define, dementia in CPRD records (e.g. codes for memory loss, or for assessment of cognitive function) were used to investigate further cases where there was uncertain agreement (neither definite agreement nor definite disagreement; see later) between HES and CPRD records of dementia as defined above.

In the postal survey of GPs we wrote asking for information about 333 study participants with a HES record of dementia before March 2008, and about 1004 study participants without a HES record of dementia by March 2008. GPs were selected to ensure a broad geographical coverage across England and, in these areas, random samples of women were selected for study. GPs were asked to complete a short questionnaire and to provide copies of relevant documents, such as letters from hospital clinics. The questionnaire asked GPs to confirm the hospital admission diagnosis of dementia (Alzheimer’s, vascular, or other); to report that they had no record of such a diagnosis; or to state if they were unable to comment, for example because of incomplete or unavailable records.

### Analyses

For comparisons with information from CPRD and from GPs, diagnoses of dementia in HES were classified as fully agreeing (evidence in primary care records to confirm a diagnosis of dementia, of any type); not agreeing (clear evidence in primary care records against a diagnosis of dementia), or of uncertain agreement (neither clear agreement nor disagreement) with primary care records. Agreement was assessed independently by at least two researchers (J.Gr. and V.B.) and discrepancies resolved by discussion. Where agreement with primary care data was uncertain, all available sources of additional information were used; and dementia mentioned on a death certificate was taken to confirm a HES record of dementia (and classified as fully agreed).

As the periods of observation in CPRD and HES differ, comparison of dementia recorded in the two databases was restricted to women with overlapping observation periods: the observation period in CPRD was required to cover at least 12 months before and 12 months after the first HES record of dementia. For these women, all available CPRD records between 1.1.1990 and 31.12.2012 were examined for dementia diagnoses.

The first mention of dementia is likely to be in primary care rather than in hospital admissions records. To estimate the time lag we calculated the difference between the date of first mention of dementia in CPRD and first mention in HES for women who had a record in both.

Age-specific rates for dementia were estimated using HES and CPRD data for the 8% of the cohort linked to CPRD. The CPRD rates used the specified periods of observation in CPRD from 1 January 1990 up to the first mention of dementia or to 31 December 2012, whichever came first. The HES rates were calculated from the date of entry into the cohort, up to whichever came first out of the first mention of dementia, death or 31 March 2011 (the last date of complete HES data). In a sensitivity analysis, age-specific rates using HES data were estimated assuming that dementia had been diagnosed 1.6 years before the first mention of dementia in the hospital records (the time difference between first mention of dementia in CPRD and first mention in HES, as described above).

## Results

**Fig. 1:**
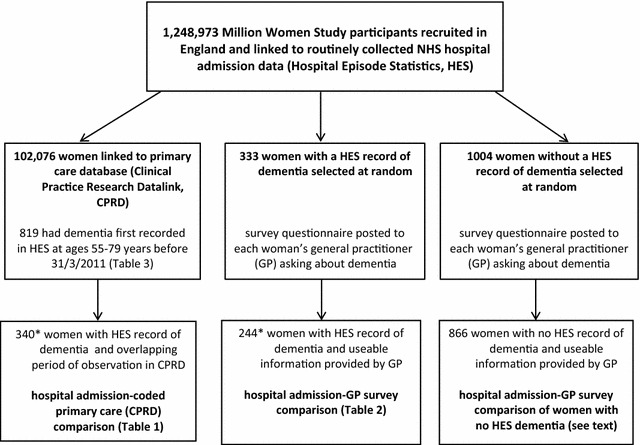
Million Women Study participants included in data comparisons. * indicates by chance, three women were included both in the coded primary care (CPRD) and in the GP survey comparison

Figure 1 summarises the study design and the number of women in each group.

HES hospital admission data were available for 1,248,973 Million Women Study participants recruited in England. Linked CPRD primary care data were available for 102,076 (8%) of the study participants who also had HES data, among whom 340 women had a HES dementia code and an overlapping period of observation in CPRD (Fig. 1).

Results of comparisons with CPRD records for these 340 women with dementia coded in HES are shown in Table 1. HES diagnoses of dementia fully agreed for 288 (85%, 95% CI 80–88%) women (278 agreed with the CPRD codes listed in Additional file 1: Code list 1 and another ten had dementia coded as cause of death). Agreement was greatest in women with more than one HES admission mentioning dementia (92%, 89–97%). For only four women (1%) did CPRD codes definitely disagree with the HES code at the time of hospital admission, e.g. the CPRD code showed an acute confusional state associated with sepsis. For the remaining 48 (14%) women agreement was uncertain; although 79% of them (38/48) had one or more of the dementia-compatible codes in CPRD listed in Additional file 1: Code list 2, such as memory loss or confusion.

**Table 1:** Comparison of dementia recorded in Hospital Episode Statistics (HES) with information from the primary care Clinical Practice Research Datalink (CPRD)

	Women with any HES record for dementia N (% of total)			Women with HES record for Alzheimer’s dementia N (% of total)			Women with HES record for vascular dementia N (% of total)			Women with HES records only for dementia, not otherwise specified N (% of total)		
		Number of admissions mentioning dementia			Number of admissions mentioning dementia			Number of admissions mentioning dementia			Number of admissions mentioning dementia	
	All	1	2+	All	1	2+	All	1	2+	All	1	2+
CPRD diagnosis fully agreed^a^	288 (85%)	93 (73%)	195 (92%)	155 (95%)	41 (93%)	114 (95%)	66 (88%)	14 (78%)	51 (90%)	65 (70%)	33 (60%)	32 (84%)
Diagnostic agreement uncertain	48 (14%)	30 (24%)	18 (8%)	9 (5%)	3 (7%)	6 (5%)	9 (12%)	4 (22%)	6 (10%)	24 (26%)	18 (33%)	6 (16%)
CPRD diagnosis disagreed	4 (1%)	4 (3%)	0	0	0	0	0	0	0	4 (4%)	4 (7%)	0
Total (=100%)	340	127	213	164	44	120	75	18	57	93	55	38

^a^All comparisons are with CPRD record of any dementia diagnosis

Table 1 also shows the results of the comparison between type of dementia coded in hospital admissions data and mention of dementia (of any type) in CPRD. Agreement was significantly greater (p < 0.05 for all comparisons) for specific HES diagnoses of Alzheimer’s disease (95%, 90–97%) or of vascular dementia (88%, 79–94%) than for a HES diagnosis of dementia, not otherwise specified (70%, 60–78%). Agreement with a CPRD record of any dementia was lowest, at 60%, for women with just one HES record of dementia, not otherwise specified.

In the comparison of HES records with information provided directly by GPs, informative responses were received for 73% (244/333) of the sample of women with a HES record of dementia (Fig. 1). No reply was received from the GP for 35 women (11%) and for 54 women (16%) the GP returned the survey form but without useable information on dementia. In most such cases the GP commented that the patient had died or moved, and the practice no longer had access to full records.

Comparisons with HES data are shown in Table 2, using the same format as in Table 1. HES diagnosis of dementia fully agreed for 208 (85%, 95% CI 80–89%) women (204 confirmed by GPs, and a further four by death certificates). As found in comparisons with CPRD records, agreement with GP reports was greatest in women with more than one HES record of dementia (94%, 90–98%). Only 9 (4%) disagreed, where the GP provided evidence that the woman did not have dementia. These included, for example, a diagnosis of dementia suspected at time of HES admission, but not confirmed on subsequent investigation; other women had diagnoses such as encephalitis, pneumonia or urinary tract infection with acute confusional state. For 27 (11%) the diagnostic comparison remained uncertain after review of all available data. The uncertain group is largely comprised of those whose GP did not confirm the HES diagnosis, but where it was not clear if the GP still had access to relevant records (if a woman dies or leaves the GP practice, including some moves to institutional care, the primary care records generally move with her).

**Table 2:** Comparison of diagnosis of dementia in Hospital Episode Statistics (HES) with primary care information provided by general practitioners (GPs)

	Women with any HES record of dementia N (% of total)			Women with HES record for Alzheimer’s dementia N (% of total)			Women with HES record for vascular dementia N (% of total)			Women with HES record only for dementia, not otherwise specified N (% of total)		
		Number of admissions mentioning dementia			Number of admissions mentioning dementia			Number of admissions mentioning dementia			Number of admissions mentioning dementia	
	All	1	2+	All	1	2+	All	1	2+	All	1	2+
GP diagnosis fully agreed^a^	208 (85%)	56 (68%)	152 (94%)	115 (94%)	19 (79%)	96 (97%)	44 (88%)	7 (78%)	37 (90%)	51 (72%)	29 (63%)	22 (88%)
Agreement uncertain	27 (11%)	20 (24%)	7 (4%)	4 (3%)	2 (8%)	2 (2%)	6 (12%)	2 (22%)	4 (10%)	15 (21%)	14 (30%)	1 (4%)
GP diagnosis disagreed	9 (4%)	6 (7%)	3 (2%)	4 (3%)	3 (13%)	1 (1%)	0	0	0	5 (7%)	3 (7%)	2 (8%)
Total (=100%)	244	82	162	123	24	99	50	9	41	71	46	25

^a^All comparisons are with GP report of any dementia diagnosis

Table 2 also shows the results of comparisons by type of dementia recorded in HES. As in Table 1, agreement between HES records and GP reports (of any dementia) was significantly greater (p < 0.05 for all comparisons) for a specific HES diagnosis of Alzheimer’s disease (94%, 89–97%) or of vascular dementia (88%, 76–94%) than for dementia, not otherwise specified (72%, 62–81%). For women with just one HES record of dementia, not otherwise specified, agreement was 63%.

GPs were also asked whether any of a randomly selected sample of 1004 women without a hospital admissions record of dementia (Fig. 1) had dementia. Informative replies were received for 86% (866/1004) of women and only one (0.1%) was reported by her GP to have dementia. No reply was received for 68 women (7%), and replies with no useable data on dementia diagnosis for the remaining 70 women (7%).

Table 3 shows estimated age-specific rates for dementia per 1000 women per year in 5 year age groups from 55–59 to 75–79 for the 102,076 study participants linked both to HES and to CPRD. There were insufficient data in the cohort to estimate rates at other ages. Dementia rates are strongly dependent on age: based on CPRD data, rates increased 80-fold between ages 55–59 and 75–79, from 0.1 to 8 per 1000 per year (Table 3A). The CPRD age-specific rates for dementia in this cohort are similar to other published rates using CPRD data [11].

**Table 3:** Age-specific rates for dementia per 1000 women per year based on data from Clinical Practice Research Datalink (CPRD) and Hospital Episode Statistics (HES)

Age (years)	(A) Using CPRD data		(B) Using HES data based on the date of first mention of dementia in HES		(C) Using HES data assuming that the date of onset of dementia is 1.6 years before the first mention of dementia in HES^a^	
	N	Rate (95% CI)	N	Rate (95% CI)	N	Rate (95% CI)
55–59	51	0.12 (0.09–0.16)	30	0.10 (0.07–0.14)	37	0.13 (0.09–0.17)
60–64	145	0.37 (0.31–0.44)	104	0.27 (0.22–0.32)	139	0.41 (0.35–0.49)
65–69	319	1.18 (1.06–1.31)	207	0.76 (0.66–0.87)	247	1.12 (0.99–1.27)
70–74	488	3.53 (3.23–3.85)	305	2.37 (2.12–2.65)	308	3.48 (3.12–3.90)
75–79	357	8.06 (7.27–8.95)	173	6.96 (5.99–8.07)	94	8.54 (6.98–10.45)

^a^See “Methods” and “Results” section

**Fig. 2:**
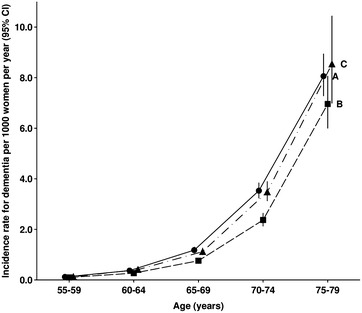
Age-specific incidence of dementia in the Million Women Study cohort, using different sources of data. *A* based on primary care data, *B* based only on hospital admissions data, *C* based on hospital admissions data, assuming a lag of 1.6 years between first diagnosis and admission to hospital, as suggested by primary care data

Age-specific rates calculated using HES data (Table 3B) are, as expected, lower than the CPRD rates (Table 3A). However, among the women with dementia recorded both in the HES and in the CPRD data, the first mention of dementia was an average of 1.6 (SD 2.6) years earlier in CPRD than in HES. In a sensitivity analysis we assumed that, for women with a HES record of dementia, the dementia had been diagnosed 1.6 years earlier; under this assumption age-specific rates are similar to those based on CPRD data (Table 3C; Fig. 2).

Of the 340 women with both a HES and a CPRD record of dementia, 64% (216) died before 31 December 2014 and dementia was mentioned on the death certificate for 37% of them (71 as the underlying cause of death and eight as a contributory cause of death).

## Discussion

Our results suggest that dementia recorded in routinely-collected coded NHS hospital admission data in England agrees well with dementia recorded in primary care. Dementia recorded in HES agreed with CPRD coded primary care records for 85% of women and disagreed for only 1%. For the remaining 14%, agreement was uncertain, although for 80% of those with uncertain agreement, less specific diagnoses such as ‘memory loss’ were recorded in CPRD. Such diagnoses were not included in our definition of dementia in CPRD, but are compatible with a diagnosis of dementia. Agreement was high, and significantly greater than for women with a record only of dementia not otherwise specified, for women with a HES code of Alzheimer’s disease (95% of whom had a CPRD record of any dementia) and of vascular dementia (88% of whom had a CPRD record of any dementia). Agreement was also excellent for women with dementia recorded in more than one HES admission (92%). Reports by GPs, who generally have access to all (not just electronically coded) information in primary care records, showed similar results.

There is limited published evidence on the reliability of routinely collected diagnoses of dementia in NHS databases. The positive predictive value of dementia coded in the General Practice Research Database (now the CPRD) has been reported to be around 80–90% [12]. We are not aware of any previous study of the reliability of diagnoses of dementia in NHS hospital admissions data. Results from studies carried out in different health care settings are difficult to compare. Positive predictive values for dementia coded in health records, compared to review of medical notes by neurologists, are reported to range from 60 to 98% [13], with equally wide variation in reported sensitivity and specificity. Primary care records can also provide relevant comparative data, because GPs hold the most comprehensive NHS health records for individuals in the UK [14]. Of particular relevance, GPs have records from specialist hospital outpatient clinics, where a confirmed diagnosis of dementia is often made. Diagnostic information from outpatient clinics is not generally available in routinely-collected hospital electronic records. Although there was excellent agreement for HES records of Alzheimer disease and of vascular dementia with records of dementia in primary care, this investigation was not designed to validate the specific subtypes of dementia.

For 16% of women in the GP survey, the GP was unable to confirm or refute a diagnosis of dementia, often because the woman had died or had moved from the practice since the date of their HES record of dementia, and historical records were no longer available. For a further 11%, no reply was received from the GP. It is possible that the missing information may have biased estimates of agreement; but results were very similar for the GP survey and for the comparison with CPRD coded records. Information available in CPRD is restricted to that collected using specific GP software systems, and may not be fully representative of all GP data in England [15]: again, the similarity of the results using coded CPRD data and using information obtained directly from a random sample of GPs suggests that this is not a major issue which would limit generalisability of the results.

Information from death certificates does not appear to reflect hospital diagnoses of dementia very closely. Two-thirds of the women with HES dementia diagnoses in the HES-CPRD comparison are known to have died subsequently, but dementia was recorded as the underlying or contributory cause of death for only a minority.

Age-specific rates of dementia for women in our cohort are similar to rates of clinically diagnosed dementia in primary care reported in other UK population-based studies [11]. Rates of clinically diagnosed dementia are, as expected, lower than rates recorded in field-based studies based on case finding [16–18], because some people with early dementia found in the case finding studies may not have been clinically diagnosed and thus would not have been recorded as having dementia in primary care data. Those diagnosed with dementia in primary care would not necessarily be admitted to hospital, and not all those admitted would have had dementia coded in their hospital records. Nevertheless, we found similar age-specific incidence rates for dementia coded in primary care (CPRD) and in hospital records, after assuming a lag of 1.6 years between first diagnosis in primary care and first admission to hospital. Also, only about 0.1% of women with no HES record of dementia were reported by their GP to have dementia. These findings suggest hospital admission data in England are not missing large numbers with dementia known in primary care.

## Conclusions

Dementia recorded in routinely collected NHS hospital admission data for women in England agrees well with primary care records of dementia assessed separately from two different sources, and is sufficiently reliable for epidemiological research.

## Additional file

**Additional file 1.** CPRD codes used to define dementia.

## Abbreviations

NHS: National Health Service
HES: Hospital Episode Statistics
CPRD: Clinical Practice Research Datalink
GP: General Practitioner
SD: standard deviation
ICD-10: 10th Revision of the International Classification of Diseases

## References

[1] Wu YT, Fratiglioni L, Matthews FE, Lobo A, Breteler MM, Skoog I, Brayne C (2015). Dementia in western Europe: epidemiological evidence and implications for policy making. Lancet Neurol..

[2] Thorvaldsson V, Macdonald SW, Fratiglioni L, Winblad B, Kivipelto M, Laukka EJ (2011). Onset and rate of cognitive change before dementia diagnosis: findings from two Swedish population-based longitudinal studies. J Int Neuropsychol Soc.

[3] Wright FL, Green J, Canoy D (2012). Vascular disease in women: comparison of diagnoses in hospital episode statistics and general practice records in England. BMC Med Res Methodol.

[4] NHS Digital. Attribution data set GP-registered populations scaled to ONS population estimates. 2011. http://digital.nhs.uk/catalogue/PUB05054. Accessed 24 Aug 16.

[5] https://www.cprd.com/. Accessed 30 Aug 2016.

[6] Herrett E, Gallagher AM, Bhaskaran K, Forbes H, Mathur R, van Staa T, Smeeth L (2015). Data resource profile: clinical practice research datalink (CPRD). Int J Epidemiol.

[7] The Million Women Study Collaborative Group (1999). The Million Women Study: design and characteristics of the study population. The Million Women Study Collaborative Group. Breast Cancer Res.

[8] Million Women Study Collaborators (2003). Breast cancer and hormone-replacement therapy in the Million Women Study. Lancet.

[9] http://www.hscic.gov.uk/. Accessed 30 Nov 2015.

[10] World Health Organization (1992). International statistical classification of diseases and related health problems.

[11] Qizilbash N, Gregson J, Johnson ME, Pearce N, Douglas I, Wing K, Evans SJ, Pocock SJ (2015). BMI and risk of dementia in two million people over two decades: a retrospective cohort study. Lancet Diabetes Endocrinol.

[12] Khan NF, Harrison SE, Rose PW (2010). Validity of diagnostic coding within the General Practice Research Database: a systematic review. Br J Gen Pract.

[13] St Germaine-Smith C, Metcalfe A, Pringsheim T, Roberts JI, Beck CA, Hemmelgarn BR (2012). Recommendations for optimal ICD codes to study neurologic conditions: a systematic review. Neurology.

[14] Herrett E, Thomas SL, Schoonen WM, Smeeth L, Hall AJ (2010). Validation and validity of diagnoses in the General Practice Research Database: a systematic review. Br J Clin Pharmacol.

[15] Kontopantelis E, Buchan I, Reeves D, Checkland K, Doran T (2013). Relationship between quality of care and choice of clinical computing system: retrospective analysis of family practice performance under the UK’s quality and outcomes framework. BMJ Open..

[16] Kosteniuk JG, Morgan DG, O’Connell ME, Kirk A, Crossley M, Teare GF, Stewart NJ, Bello-Haas VD, Forbes DA, Innes A, Quail JM (2015). Incidence and prevalence of dementia in linked administrative health data in Saskatchewan, Canada: a retrospective cohort study. BMC Geriatr.

[17] Matthews F, Brayne C, Medical Research Council Cognitive Function and Ageing Study Investigators (2005). The incidence of dementia in England and Wales: findings from the five identical sites of the MRC CFA Study. PLoS Med.

[18] Russell P, Banerjee S, Watt J, Adleman R, Agoe B, Burnie N (2013). Improving the identification of people with dementia in primary care: evaluation of the impact of primary care dementia coding guidance on identified prevalence. BMJ Open.

