# Supplementary material for: Comparison of dementia recorded in routinely collected hospital admission data in England with dementia recorded in primary care
Source: Emerg Themes Epidemiol. 2016 Oct 28;13:11. doi: 10.1186/s12982-016-0053-z (PMC5084368; doi:10.1186/s12982-016-0053-z)
Supplement: Supplementary file 1 — Additional file 1. CPRD codes used to define dementia. [file 12982_2016_53_MOESM1_ESM.docx]

**Additional file 1. CPRD codes used to define dementia**

**Code list 1: CPRD Read codes and terms for dementia**

| **Read Code** | **Read Term** |
| --- | --- |
| 1461.00 | H/O: dementia |
| 3AE..00 | Global deterioration scale: assessment of prim deg dementia |
| 3AE1.00 | GDS level 2 – very mild cognitive decline |
| 3AE2.00 | GDS level 3 – mild cognitive decline |
| 3AE3.00 | GDS level 4 – moderate cognitive decline |
| 3AE4.00 | GDS level 5 – moderately severe cognitive decline |
| 3AE5.00 | GDS level 6 – severe cognitive decline |
| 3AE6.00 | GDS level 7 – very severe cognitive decline |
| 66h..00 | Dementia monitoring |
| 6AB..00 | Dementia annual review |
| 8Hla.00 | Referral to dementia care advisor |
| 9Ou1.00 | Dementia monitoring first letter |
| 9Ou2.00 | Dementia monitoring second letter |
| 9Ou3.00 | Dementia monitoring third letter |
| 9Ou4.00 | Dementia monitoring verbal invite |
| 9Ou5.00 | Dementia monitoring telephone invite |
| 9hD..00 | Exception reporting: dementia quality indicators |
| 9hD0.00 | Excepted from dementia quality indicators: Patient unsuitabl |
| 9hD1.00 | Excepted from dementia quality indicators: Informed dissent |
| C251.11 | Wernicke’s encephalopathy |
| C253.00 | Wernicke’s encephalopathy |
| E00..11 | Senile dementia |
| E00..12 | Senile/presenile dementia |
| E000.00 | Uncomplicated senile dementia |
| E001.00 | Presenile dementia |
| E001000 | Uncomplicated presenile dementia |
| E001100 | Presenile dementia with delirium |
| E001200 | Presenile dementia with paranoia |
| E001300 | Presenile dementia with depression |
| E001z00 | Presenile dementia NOS |
| E002.00 | Senile dementia with depressive or paranoid features |
| E002000 | Senile dementia with paranoia |
| E002100 | Senile dementia with depression |
| E002z00 | Senile dementia with depressive or paranoid features NOS |
| E003.00 | Senile dementia with delirium |
| E004.00 | Arteriosclerotic dementia |
| E004.11 | Multi infarct dementia |
| E004000 | Uncomplicated arteriosclerotic dementia |
| E004100 | Arteriosclerotic dementia with delirium |
| E004200 | Arteriosclerotic dementia with paranoia |
| E004300 | Arteriosclerotic dementia with depression |
| E004z00 | Arteriosclerotic dementia NOS |
| E011000 | Korsakov’s alcoholic psychosis |
| E011100 | Korsakov’s alcoholic psychosis with peripheral neuritis |
| E011200 | Wernicke-Korsakov syndrome |
| E012.00 | Other alcoholic dementia |
| E012.11 | Alcoholic dementia NOS |
| E012000 | Chronic alcoholic brain syndrome |
| E040.11 | Korsakoff's non-alcoholic psychosis |

| **Read Code** | **Read Term** |
| --- | --- |
| E041.00 | Dementia in conditions EC |
| Eu00.00 | [X]Dementia in Alzheimer's disease |
| Eu00000 | [X]Dementia in Alzheimer's disease with early onset |
| Eu00011 | [X]Presenile dementia,Alzheimer's type |
| Eu00012 | [X]Primary degen dementia, Alzheimer's type, presenile onset |
| Eu00013 | [X]Alzheimer's disease type 2 |
| Eu00100 | [X]Dementia in Alzheimer's disease with late onset |
| Eu00111 | [X]Alzheimer's disease type 1 |
| Eu00112 | [X]Senile dementia,Alzheimer's type |
| Eu00113 | [X]Primary degen dementia of Alzheimer's type, senile onset |
| Eu00200 | [X]Dementia in Alzheimer's dis, atypical or mixed type |
| Eu00z00 | [X]Dementia in Alzheimer's disease, unspecified |
| Eu00z11 | [X]Alzheimer's dementia unspec |
| Eu01.00 | [X]Vascular dementia |
| Eu01.11 | [X]Arteriosclerotic dementia |
| Eu01000 | [X]Vascular dementia of acute onset |
| Eu01100 | [X]Multi-infarct dementia |
| Eu01111 | [X]Predominantly cortical dementia |
| Eu01200 | [X]Subcortical vascular dementia |
| Eu01300 | [X]Mixed cortical and subcortical vascular dementia |
| Eu01y00 | [X]Other vascular dementia |
| Eu01z00 | [X]Vascular dementia, unspecified |
| Eu02.00 | [X]Dementia in other diseases classified elsewhere |
| Eu02000 | [X]Dementia in Pick's disease |
| Eu02100 | [X]Dementia in Creutzfeldt-Jakob disease |
| Eu02200 | [X]Dementia in Huntington's disease |
| Eu02300 | [X]Dementia in Parkinson's disease |
| Eu02400 | [X]Dementia in human immunodef virus [HIV] disease |
| Eu02500 | [X]Lewy body dementia |
| Eu02y00 | [X]Dementia in other specified diseases classif elsewhere |
| Eu02z00 | [X] Unspecified dementia |
| Eu02z11 | [X] Presenile dementia NOS |
| Eu02z13 | [X] Primary degenerative dementia NOS |
| Eu02z14 | [X] Senile dementia NOS |
| Eu02z16 | [X] Senile dementia, depressed or paranoid type |
| Eu03.11 | [X]Korsakov's psychosis, nonalcoholic |
| Eu04100 | [X]Delirium superimposed on dementia |
| Eu10611 | [X]Korsakov's psychosis, alcohol induced |
| Eu10711 | [X]Alcoholic dementia NOS |
| Eu10712 | [X]Chronic alcoholic brain syndrome |
| F110.00 | Alzheimer's disease |
| F110000 | Alzheimer's disease with early onset |
| F110100 | Alzheimer's disease with late onset |
| F111.00 | Pick's disease |
| F112.00 | Senile degeneration of brain |
| F116.00 | Lewy body disease |
| ZS78D00 | Wernicke's dysphasia |
| ZS78D13 | Wernicke's aphasia |

**Additional file 1 (continued)**

**Code List 1: CPRD codes for Dementia Medicinal Products [BNF 4.1.1]**

| **Gemscript Code** | **Medicinal Product Name** |
| --- | --- |
| 84654020 | Aricept 10mg tablets (Eisai Ltd) |
| 84653020 | Aricept 5mg tablets (Eisai Ltd) |
| 05435020 | Aricept 5mg tablets (Waymade Healthcare Plc) |
| 92625020 | Aricept Evess 10mg orodispersible tablets (Eisai Ltd) |
| 92623020 | Aricept Evess 5mg orodispersible tablets (Eisai Ltd) |
| 41262020 | Donepezil 10mg orodispersible tablets |
| 41263020 | Donepezil 10mg orodispersible tablets (Consilient Health Ltd) |
| 92621020 | Donepezil 10mg orodispersible tablets sugar free |
| 84649020 | Donepezil 10mg tablets |
| 39844020 | Donepezil 10mg tablets (A A H Pharmaceuticals Ltd) |
| 41264020 | Donepezil 5mg orodispersible tablets |
| 92619020 | Donepezil 5mg orodispersible tablets sugar free |
| 84648020 | Donepezil 5mg tablets |
| 42200020 | Donepezil 5mg tablets (Zentiva) |
| 85758020 | Galantamine 12mg tablets |
| 89202020 | Galantamine 16mg modified-release capsules |
| 76502020 | Galantamine 20mg/5ml oral solution sugar free |
| 89204020 | Galantamine 24mg modified-release capsules |
| 85756020 | Galantamine 4mg tablets |
| 89200020 | Galantamine 8mg modified-release capsules |
| 85757020 | Galantamine 8mg tablets |
| 00321021 | Galsya XL 24mg capsules (Consilient Health Ltd) |
| 00317021 | Galsya XL 8mg capsules (Consilient Health Ltd) |
| 45236020 | Gatalin XL 16mg capsules (Aspire Pharma Ltd) |
| 45237020 | Gatalin XL 24mg capsules (Aspire Pharma Ltd) |
| 45238020 | Gatalin XL 8mg capsules (Aspire Pharma Ltd) |
| 76137020 | Reminyl 12mg tablets (Shire Pharmaceuticals Ltd) |
| 76135020 | Reminyl 4mg tablets (Shire Pharmaceuticals Ltd) |
| 86236020 | Reminyl 4mg/ml oral solution (Shire Pharmaceuticals Ltd) |
| 76136020 | Reminyl 8mg tablets (Shire Pharmaceuticals Ltd) |
| 89208020 | Reminyl XL 16mg capsules (Shire Pharmaceuticals Ltd) |
| 89210020 | Reminyl XL 24mg capsules (Shire Pharmaceuticals Ltd) |
| 89206020 | Reminyl XL 8mg capsules (Shire Pharmaceuticals Ltd) |
| 10541020 | Ebixa 10mg tablets (Doncaster Pharmaceuticals Ltd) |
| 84771020 | Ebixa 10mg tablets (Lundbeck Ltd) |
| 95627020 | Ebixa 20mg tablets (Lundbeck Ltd) |
| 55435020 | Ebixa 5mg/pump oral solution (Lundbeck Ltd) |
| 95639020 | Ebixa tablets treatment initiation pack (Lundbeck Ltd) |
| 80100020 | Memantine 10mg tablets |
| 80102020 | Memantine 10mg/ml oral solution sugar free |
| 95625020 | Memantine 20mg tablets |
| 95635020 | Memantine 5mg+10mg+15mg+20mg Tablet |
| 80919020 | Exelon 1.5mg capsules (Novartis Pharmaceuticals UK Ltd) |
| 16263021 | Exelon 13.3 mg/24hours transdermal patches (Novartis Pharmaceuticals UK Ltd) |
| 77547020 | Exelon 2mg/ml oral solution (Novartis Pharmaceuticals UK Ltd) |
| 80920020 | Exelon 3mg capsules (Novartis Pharmaceuticals UK Ltd) |

| **Gemscript Code** | **Medicinal Product Name** |
| --- | --- |
| 80921020 | Exelon 4.5mg capsules (Novartis Pharmaceuticals UK Ltd) |
| 05472020 | Exelon 4.5mg capsules (Waymade Healthcare Plc) |
| 94174020 | Exelon 4.6mg/24hours transdermal patches (Novartis Pharmaceuticals UK Ltd) |
| 80912020 | Exelon 6mg capsules (Novartis Pharmaceuticals UK Ltd) |
| 94176020 | Exelon 9.5mg/24hours transdermal patches (Novartis Pharmaceuticals UK Ltd) |
| 58075020 | Rivastigmine 1.5mg capsules |
| 16262021 | Rivastigmine 13.3mg/24hours transdermal patches |
| 43884020 | Rivastigmine 2mg/ml oral solution |
| 77544020 | Rivastigmine 2mg/ml oral solution sugar free |
| 58076020 | Rivastigmine 3mg capsules |
| 41393020 | Rivastigmine 3mg capsules (Dr Reddy's Laboratories (UK) Ltd) |
| 58077020 | Rivastigmine 4.5mg capsules |
| 94170020 | Rivastigmine 4.6mg/24hours transdermal patches |
| 76950020 | Rivastigmine 6mg capsules |
| 94172020 | Rivastigmine 9.5mg/24hours transdermal patches |
| 16508021 | Rivatev 4.6mg/24hours transdermal patches (Teva UK Ltd) |
| 16510021 | Rivatev 9.5mg/24hours transdermal patches (Teva UK Ltd) |
| 15913021 | Voleze 9.5mg/24hours transdermal patches (Focus Pharmaceuticals Ltd) |

**Additional file 1 continued**

**Code list 2: Additional Read terms compatible with, but not defining, dementia**

| **Read Code** | **Read Term** |
| --- | --- |
| 1479.00 | H/O: disturbance of consciousness |
| 147F.00 | History of Parkinson's disease |
| 14Od.00 | At risk of dementia |
| 1B1A.00 | Memory loss - amnesia |
| 1B1A.11 | Amnesia symptom |
| 1B1A.12 | Memory loss symptom |
| 1B1A.13 | Memory disturbance |
| 1B1A000 | Temporary loss of memory |
| 1B1A100 | Short-term memory loss |
| 1B6..00 | Disturbance of consciousness |
| 1BN..00 | Wandering |
| 1BN0.00 | Wanders during the day |
| 1BN2.00 | Wanders during the day and at night |
| 1JA2.00 | Suspected dementia |
| 2232.00 | O/E - mentally confused |
| 2232.11 | O/E - confused |
| 2233.00 | O/E - delirious |
| 2841.11 | Confusion |
| 2JR..00 | Lack mental capacity make decision Mental Capacity Act 2005 |
| 388V.00 | Mini mental state score |
| 388m.00 | Mini-mental state examination |
| 388m.11 | MMSE score |
| 38Dv.00 | GPCOG - general practitioner assessment of cognition |
| 38Dv000 | GPCOG (GP assessment of cognition) patient examination |
| 38Dv100 | GPCOG (GP assessment of cognition) informant interview |
| 3A...11 | Memory assessment |
| 3A...12 | Dementia assessment |
| 3A1..00 | Memory: own age |
| 3A11.00 | Memory: own age known |
| 3A20.00 | Memory: present time not known |
| 3A21.00 | Memory: present time known |
| 3A3..00 | Memory: present place |
| 3A30.00 | Memory: present place not knwn |
| 3A31.00 | Memory: present place known |
| 3A4..00 | Memory: present year |
| 3A40.00 | Memory: present year not known |
| 3A41.00 | Memory: present year known |
| 3A51.00 | Memory: own DOB known |
| 3A60.00 | Memory: present month not knwn |
| 3A61.00 | Memory: present month known |
| 3A71.00 | Memory: important event known |
| 3A80.00 | Memory: import.person not knwn |
| 3A81.00 | Memory: important person known |
| 3A9..00 | Memory: count down |
| 3A91.00 | Memory: count down unsuccess. |
| 3AA..00 | Memory: address recall |

| **Read Code** | **Read Term** |
| --- | --- |
| 3AA0.00 | Memory: address recall success |
| 3AD..00 | Dementia test |
| 3AD1.00 | Ten item dementia test |
| 3AD2.00 | Thirty seven item dementia test |
| 3AD3.00 | Six item cognitive impairment test |
| 3AF..00 | Addenbrooke's cognitive examination revised |
| 8HTY.00 | Referral to memory clinic |
| 9Ou..00 | Dementia monitoring administration |
| E....00 | Mental disorders |
| E010.12 | Delirium tremens |
| E011.00 | Alcohol amnestic syndrome |
| E030.00 | Acute confusional state |
| E030.11 | Delirium - acute organic |
| E2A1000 | Mild memory disturbance |
| E2A2.00 | Post-concussion syndrome |
| E2A2.12 | Post-head injury syndrome |
| Eu02z15 | [X] Senile psychosis NOS |
| Eu04.11 | [X]Acute / subacute brain syndrome |
| Eu04.12 | [X]Acute / subacute confusional state, nonalcoholic |
| Eu04z00 | [X]Delirium, unspecified |
| Eu05.00 | [X]Oth mental disorder brain damag/dysfunction/physical disr |
| Eu05300 | [X]Organic mood [affective] disorders |
| Eu05700 | [X]Mild cognitive disorder |
| Eu06200 | [X]Postconcussional syndrome |
| Eu06212 | [X]Post-traumatic brain syndrome |
| Ez...00 | Mental disorders NOS |
| F11..00 | Other cerebral degenerations |
| F118.00 | Frontotemporal degeneration |
| F11x000 | Cerebral degeneration due to alcoholism |
| F11x011 | Alcoholic encephalopathy |
| F11x700 | Cerebral degeneration due to Jakob - Creutzfeldt disease |
| F11x900 | Cerebral degeneration in Parkinson's disease |
| F11z.00 | Cerebral degeneration NOS |
| F11z.11 | Cerebral atrophy |
| R009.00 | [D]Confusion |
| Z7CEH00 | Memory impairment |
| ZR16.00 | Abbreviated mental test |
| ZR16.11 | AMT - Abbreviated mental test |
| ZR1H.00 | Allen cognitive level screening tool |
| ZR2X.11 | Memory concentration test |
| ZR3V.12 | CDR - Clinical dementia rating scale |
| ZR3a.00 | Cognitions questionnaire |
| ZRBg.00 | Everyday memory questionnaire |
| ZRa2.00 | Microcog - assessment of cognitive function |
| ZRaA.11 | MMSE - Mini-mental state examination |
| ZRaA200 | Modified mini-mental state examination |
